# Supplementary material for: Quantitative proteomics and in-cell cross-linking reveal cellular reorganisation during early neuronal differentiation of SH-SY5Y cells
Source: Commun Biol. 2022 Jun 7;5:551. doi: 10.1038/s42003-022-03478-7 (PMC9174471; doi:10.1038/s42003-022-03478-7)
Supplement: Supplementary file 2 — Supplementary Information [file 42003_2022_3478_MOESM2_ESM.pdf]

Quantitative proteomics and in-cell cross-linking reveal cellular reorganisation during early neuronal differentiation of SH-SY5Y cells

Marie Barth[1], Alicia Toto Nienguesso[2], Anne Navarrete Santos[2] and Carla Schmidt[1]*

[1]Interdisciplinary Research Center HALOmem, Institute of Biochemistry and Biotechnology, Charles Tanford Protein Center, Martin Luther University Halle-Wittenberg, Halle, Germany

[2]Institute of Anatomy and Cell Biology, Faculty of Medicine, Martin Luther University Halle-Wittenberg, Halle, Germany

*correspondence: carla.schmidt@biochemtech.uni-halle.de

Supplementary Figures

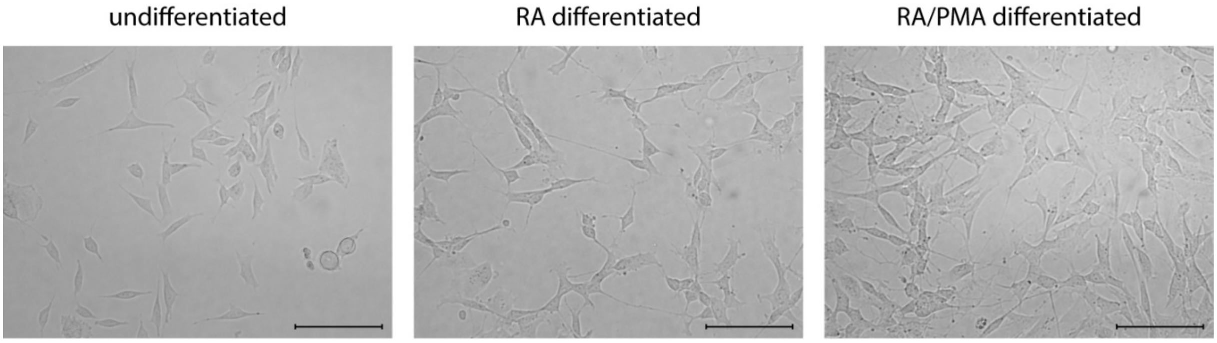

Supplementary Figure 1: SH-SY5Y cells.

Cells grown under standard conditions (undifferentiated), after 5 days of RA treatment (RA-differentiated) and after 6 days of RA/PMA treatment (RA/PMA-differentiated) are shown (see **Methods** section for details). Scale bar: 100 µm.

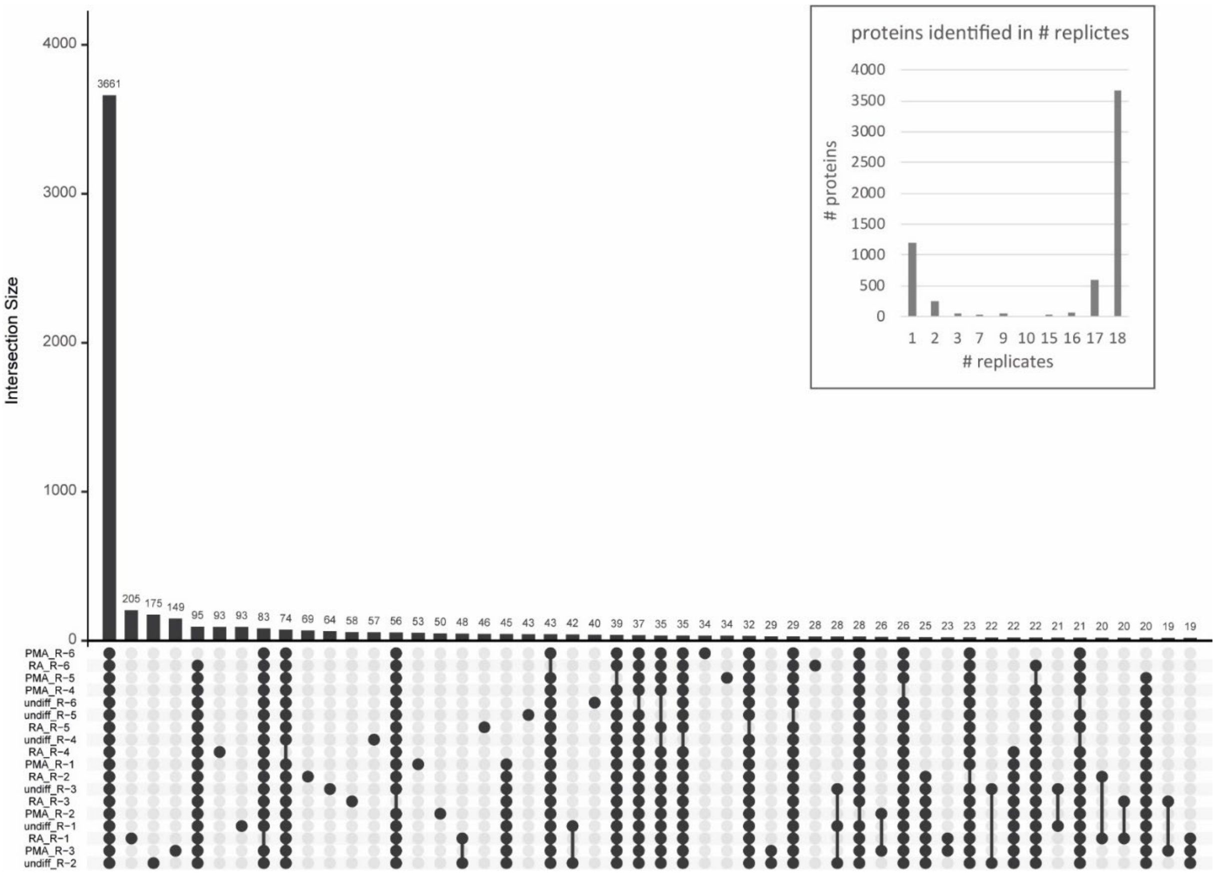

Supplementary Figure 2: Protein identification.

The number of identified proteins in the respective replicates is given (n=6 for each cell culture condition). 3661 proteins were identified in all 18 samples. The number of proteins identified in #replicates are shown (box). Abbreviations: Undiff, undifferentiated; RA, RA-differentiated, PMA, RA/PMA-differentiated, R, replicate.

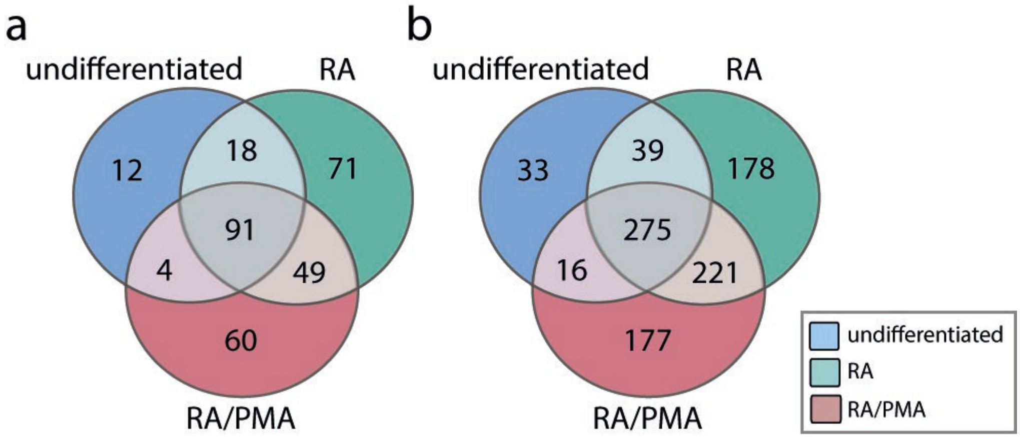

Supplementary Figure 3

SH-SY5Y cells were cross-linked using formaldehyde. **a)** Inter-molecular cross-links identified in undifferentiated (blue) as well as RA- (green) and RA/PMA- (red) differentiated cells. **b)** Intra-molecular cross-links identified in undifferentiated (blue) as well as RA- (green) and RA/PMA- (red) differentiated cells.

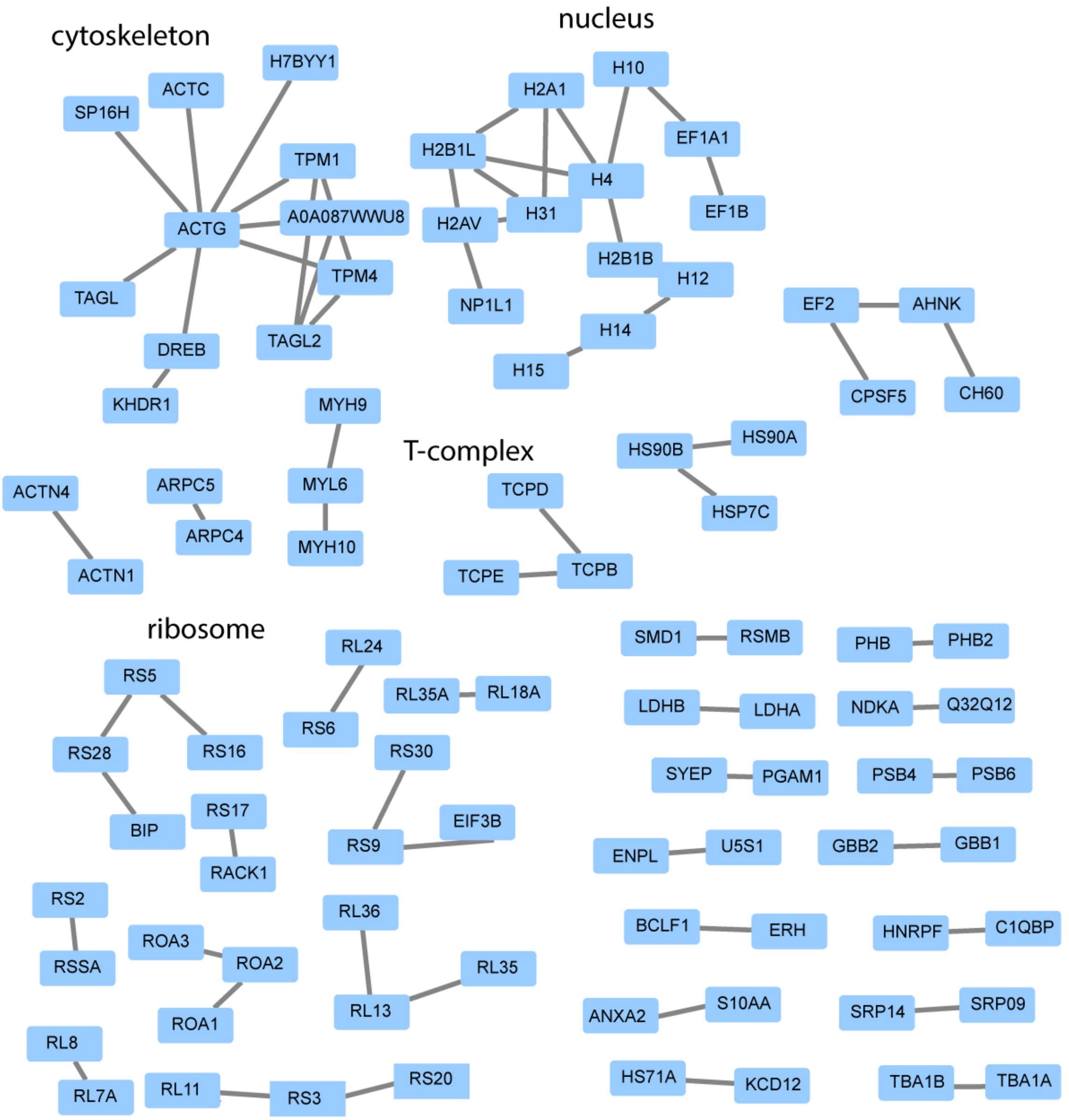

Supplementary Figure 4: Inter-molecular cross-links identified in undifferentiated SH-SY5Y cells.

Interaction network of proteins that were found to be cross-linked in undifferentiated cells. Proteins are indicated by boxes; interactions are indicated by lines. Gene names of the proteins are given. Note that information on residue specific cross-linked sites is not contained.

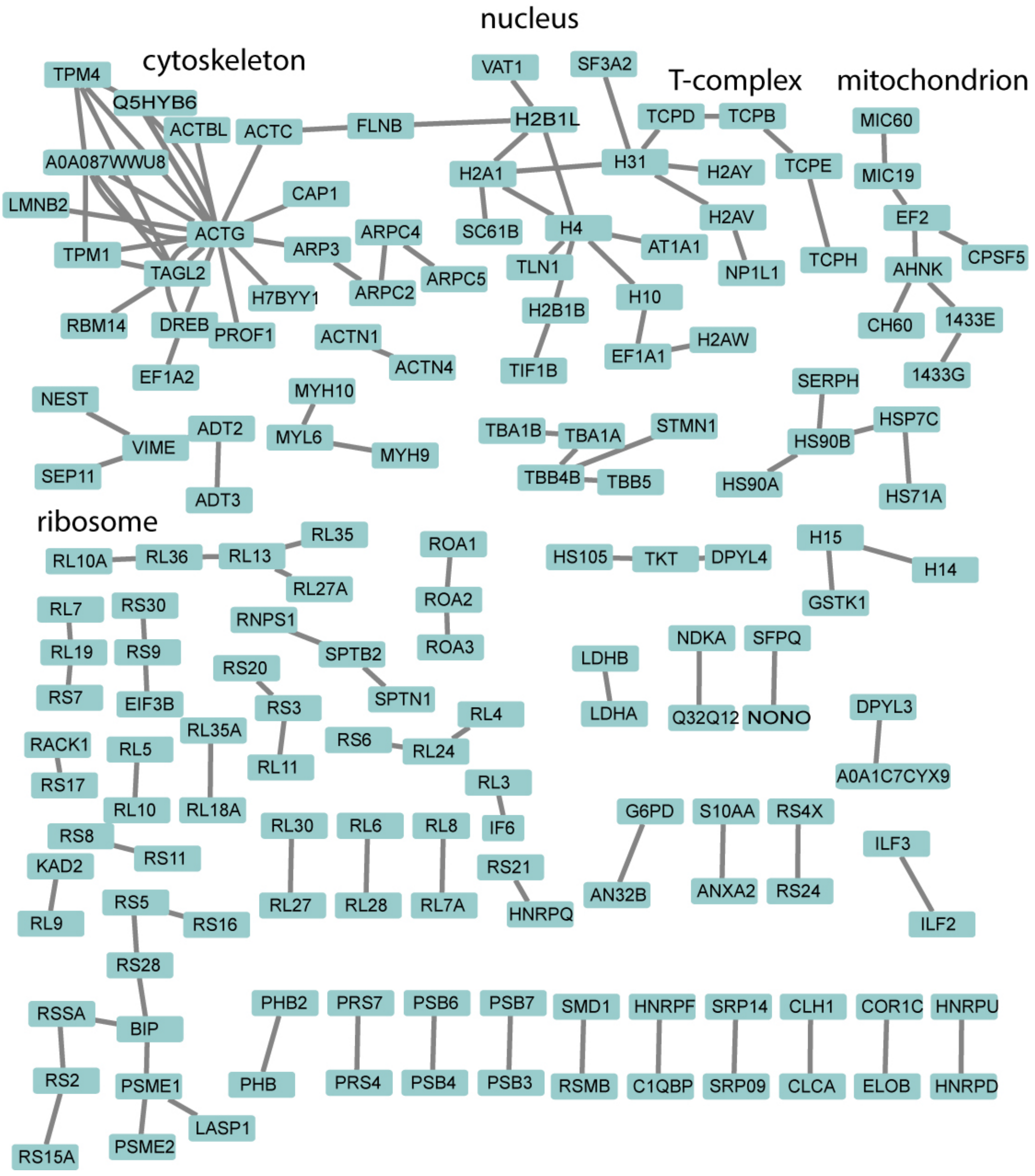

Supplementary Figure 5: Inter-molecular cross-links identified in RA-differentiated SH-SY5Y cells.

Interaction network of proteins that were found to be cross-linked in RA-differentiated cells. Proteins are indicated by boxes; interactions are indicated by lines. Gene names of the proteins are given. Note that information on residue specific cross-linked sites is not contained.

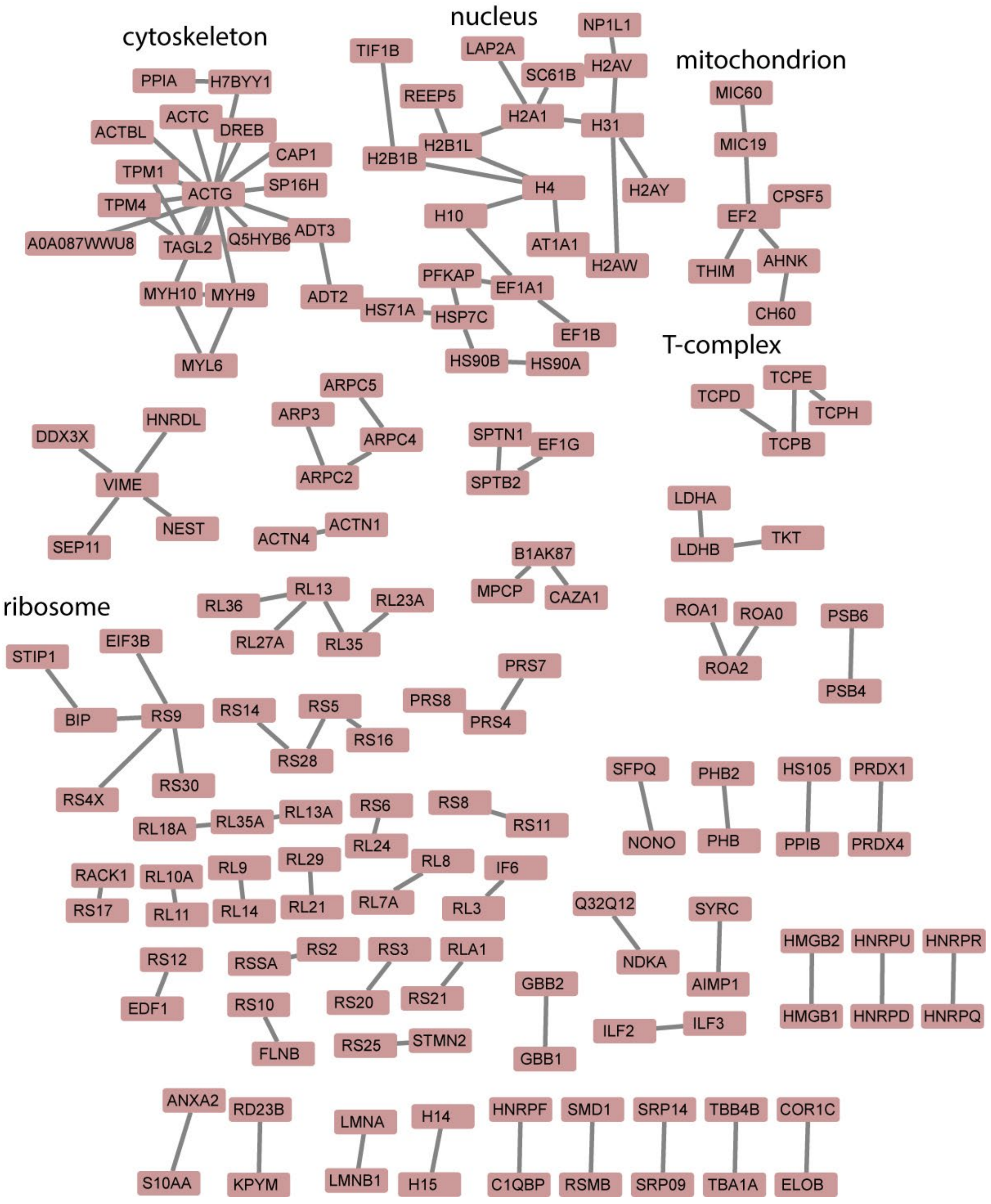

Supplementary Figure 6: Inter-molecular cross-links identified in RA/PMA-differentiated SH-SY5Y cells.

Interaction network of proteins that were found to be cross-linked in RA/PMA-differentiated cells. Proteins are indicated by boxes; interactions are indicated by lines. Gene names of the proteins are given. Note that information on residue specific cross-linked sites is not contained.

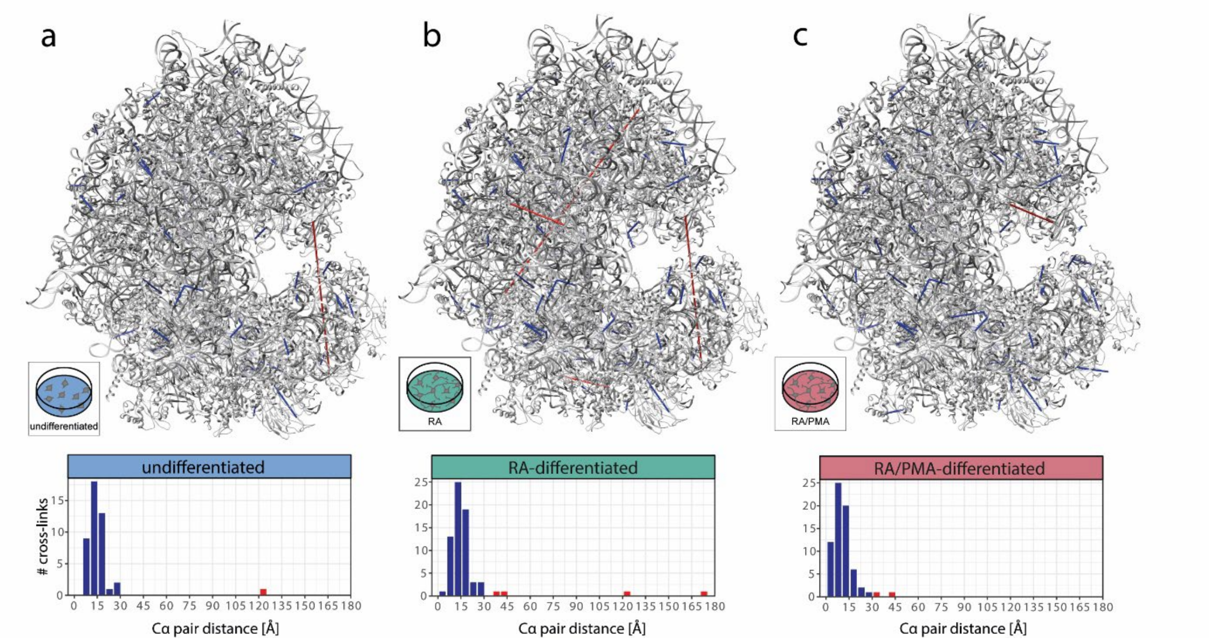

Supplementary Figure 7: Inter- and intra-molecular cross-links of ribosomal proteins.

Identified ribosomal inter- and intra-molecular cross-links are visualised on the high-resolution structure of the ribosome (PDB: 4UG0). The number of cross-links with Cα-distances < 30 Å (blue) or > 30 Å (red) is given for each cell culture condition.

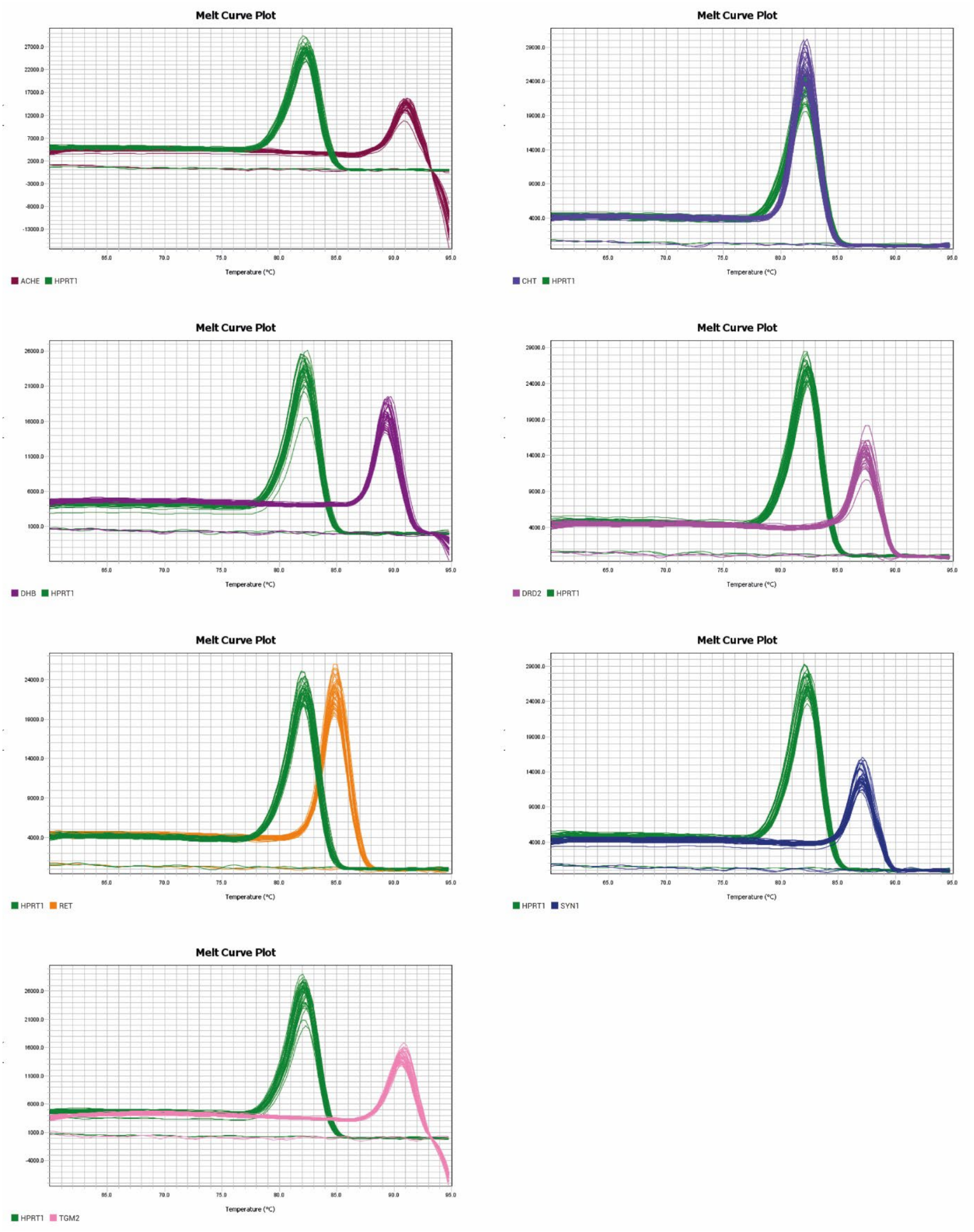

Supplementary Figure 8: Melting curve plots

The graphs represent melting curve plots of specific target genes (see legend for details). For each experiment 6 biological replicates of undifferentiated, RA- and RA/PMA-differentiated cells were measured in technical duplicates. In no template controls no amplification was observed.
