# Supplementary material for: Quantitative proteomics and in-cell cross-linking reveal cellular reorganisation during early neuronal differentiation of SH-SY5Y cells
Source: Commun Biol. 2022 Jun 7;5:551. doi: 10.1038/s42003-022-03478-7 (PMC9174471; doi:10.1038/s42003-022-03478-7)
Supplement: Supplementary file 3 — Description of Additional Supplementary Files [file 42003_2022_3478_MOESM3_ESM.pdf]

Description of Additional Supplementary Files

File name: Supplementary Data 1
Description: Relative quantification of proteins with a specific subcellular localisation. Data is plotted in Figure 2.

File name: Supplementary Data 2
Description: Proteome analysis and enriched KEGG pathways. Data is plotted in Figure 3.

File name: Supplementary Data 3
Description: qPCR. Data is plotted in Figure 4 and Supplementary Figure 8.

File name: Supplementary Data 4
Description: Cross-links in SH-SY5Y cells. Page 7 of 16 Data is plotted in Figure 5-7 and Supplementary Figures 3-7.
