# Supplementary material for: Quantitative proteomics and in-cell cross-linking reveal cellular reorganisation during early neuronal differentiation of SH-SY5Y cells
Source: Commun Biol. 2022 Jun 7;5:551. doi: 10.1038/s42003-022-03478-7 (PMC9174471; doi:10.1038/s42003-022-03478-7)
Supplement: Supplementary file 8 — Reporting Summary [file 42003_2022_3478_MOESM8_ESM.pdf]

# nature portfolio

Corresponding author(s): Carla Schmidt
Last updated by author(s): Mar 30, 2022

# Reporting Summary

Nature Portfolio wishes to improve the reproducibility of the work that we publish. This form provides structure for consistency and transparency in reporting. For further information on Nature Portfolio policies, see our Editorial Policies and the Editorial Policy Checklist.

## Statistics

For all statistical analyses, confirm that the following items are present in the figure legend, table legend, main text, or Methods section.

| n/a | Confirmed | |
|---|---|---|
| ☐ | ☒ | The exact sample size ($n$) for each experimental group/condition, given as a discrete number and unit of measurement |
| ☐ | ☒ | A statement on whether measurements were taken from distinct samples or whether the same sample was measured repeatedly |
| ☐ | ☒ | The statistical test(s) used AND whether they are one- or two-sided<br>*Only common tests should be described solely by name; describe more complex techniques in the Methods section.* |
| ☒ | ☐ | A description of all covariates tested |
| ☒ | ☐ | A description of any assumptions or corrections, such as tests of normality and adjustment for multiple comparisons |
| ☐ | ☒ | A full description of the statistical parameters including central tendency (e.g. means) or other basic estimates (e.g. regression coefficient) AND variation (e.g. standard deviation) or associated estimates of uncertainty (e.g. confidence intervals) |
| ☐ | ☒ | For null hypothesis testing, the test statistic (e.g. $F$, $t$, $r$) with confidence intervals, effect sizes, degrees of freedom and $P$ value noted<br>*Give P values as exact values whenever suitable.* |
| ☒ | ☐ | For Bayesian analysis, information on the choice of priors and Markov chain Monte Carlo settings |
| ☒ | ☐ | For hierarchical and complex designs, identification of the appropriate level for tests and full reporting of outcomes |
| ☒ | ☐ | Estimates of effect sizes (e.g. Cohen's $d$, Pearson's $r$), indicating how they were calculated |

*Our web collection on statistics for biologists contains articles on many of the points above.*

## Software and code

Policy information about availability of computer code

| | |
|---|---|
| Data collection | Vendor specific software for MS instrumentation used (Xcalibur v4.2.47). For qPCR analysis, the Quant Studio 3™ Real Time System and the QuantStudioTM Design & Analysis Software v1.5.2 (Thermo Scientific) was used. |
| Data analysis | Open source software used for data analysis: MaxQuant (version 1.6.17.0), RStudio (version 1.2.1335) using R (version 4.0.2) with artMS (version 1.9.1) (http://artms.org) and MSstats (version 3.22.0). Cross-linking analysis was performed using 'Formaldehyde Cross-link Analyser' (Tayri-Wilk, 2020) and was further analysed using the web-based venn-tool (http://bioinformatics.psb.ugent.be/webtools/Venn/) and Cytoscape (version 3.8.2) using the Cytoscape app XlinkCyNET (version 1.2.5) as well as UCSF Chimera (version 1.15) and the software tool Xlink Analyzer (version 1.1.4). Files for data analysis are deposited to the ProteomeXchange Consortium (www.proteomexchange.org) via the PRIDE partner repository with the dataset identifier PXD031054. For qPCR data analysis, QuantStudioTM Design & Analysis Software v1.5.2 (Thermo Scientific) was used. |

For manuscripts utilizing custom algorithms or software that are central to the research but not yet described in published literature, software must be made available to editors and reviewers. We strongly encourage code deposition in a community repository (e.g. GitHub). See the Nature Portfolio guidelines for submitting code & software for further information.

## Data

Policy information about availability of data

All manuscripts must include a data availability statement. This statement should provide the following information, where applicable:

- Accession codes, unique identifiers, or web links for publicly available datasets
- A description of any restrictions on data availability
- For clinical datasets or third party data, please ensure that the statement adheres to our policy

All MS raw files and the corresponding results files including databases were deposited to the ProteomeXchange Consortium (www.proteomexchange.org) via the PRIDE partner repository with the dataset identifier PXD031054.

# Field-specific reporting

Please select the one below that is the best fit for your research. If you are not sure, read the appropriate sections before making your selection.

☒ Life sciences ☐ Behavioural & social sciences ☐ Ecological, evolutionary & environmental sciences

For a reference copy of the document with all sections, see nature.com/documents/nr-reporting-summary-flat.pdf

# Life sciences study design

All studies must disclose on these points even when the disclosure is negative.

| | |
|---|---|
| Sample size | No statistical method was used for sample size calculation. For all experiments at least 3 biological replicates were used. An FDR was applied during database searches. Cross-links were filtered by score. |
| Data exclusions | No data excluded. |
| Replication | Biological and technical replicates were performed. Proteomics: 6 biological replicates, Cross-linking: 3 biological replicates. qPCR: 6 biological replicates, 2 technical replicates each |
| Randomization | Randomization is not relevant to the study because only one sample per experiment was used. |
| Blinding | Blinding was not relevant to the study because the samples are known and no subjective qualitative results were reported. |

# Reporting for specific materials, systems and methods

We require information from authors about some types of materials, experimental systems and methods used in many studies. Here, indicate whether each material, system or method listed is relevant to your study. If you are not sure if a list item applies to your research, read the appropriate section before selecting a response.

## Materials & experimental systems

| n/a | Involved in the study | |
|---|---|---|
| ☒ | ☐ | Antibodies |
| ☐ | ☒ | Eukaryotic cell lines |
| ☒ | ☐ | Palaeontology and archaeology |
| ☒ | ☐ | Animals and other organisms |
| ☒ | ☐ | Human research participants |
| ☒ | ☐ | Clinical data |
| ☒ | ☐ | Dual use research of concern |

## Methods

| n/a | Involved in the study | |
|---|---|---|
| ☒ | ☐ | ChIP-seq |
| ☒ | ☐ | Flow cytometry |
| ☒ | ☐ | MRI-based neuroimaging |

## Eukaryotic cell lines

Policy information about cell lines

| | |
|---|---|
| Cell line source(s) | The cell line SH-SY5Y was obtained from Deutsche Sammlung von Mikroorganismen und Zellkulturen (DSMZ). |
| Authentication | No specific authentication was performed, because the vendor DSMZ validated authenticity of fingerprint by multiplex PCR of minisatellite markers that revealed a unique DNA profile, species originally as human with IEF of AST, MDH and later by STR-fingerprinting and cytogenetics of human near-diploid karyotype with 1.8% polyploidy - 46/47(42-48)<2n>X/XX, +7, ins(1)(q32q11q43), add(9)(q34), der(22)t(?17;22)(q22;q13) resembled published karyotype. |

| | |
|---|---|
| Mycoplasma contamination | No specific mycoplasma test was performed, because the cell line was directly used after purchase. The vendor DSMZ test for mycoplasma by negative in DAPI, microbiological culture, RNA hybridization and PCR assays. |
| Commonly misidentified lines (See ICLAC register) | The cell line SH-SY5Y is not a commonly misidentified cell line. |
